# Accuracy of acetabular component alignment with surgical guidance systems during hip arthroplasty

**DOI:** 10.1051/sicotj/2023010

**Published:** 2023-05-05

**Authors:** Joshua Xu, Ewout S. Veltman, Yuan Chai, William L. Walter

**Affiliations:** 1 University of Sydney Sydney New South Wales Australia; 2 Department of Orthopaedic and Trauma Surgery, Royal North Shore Hospital St. Leonards New South Wales Australia; 3 Tom Reeve Academic Clinic, Ground Floor Kolling Building 10 Westbourne Street St Leonards NSW 2065 Australia

**Keywords:** Navigation, Hip arthroplasty, Optical navigation, Inertial navigation, Acetabular positioning

## Abstract

*Background*: Navigation in total hip arthroplasty has been shown to improve acetabular positioning and can decrease the incidence of mal-positioned acetabular components. This study aimed to assess two surgical guidance systems by comparing intra-operative measurements of acetabular component inclination and anteversion with a post-operative CT scan. *Methods*: We prospectively collected intra-operative navigation data from 102 hips receiving conventional THA or hip resurfacing arthroplasty through either a direct anterior or posterior approach. Two guidance systems were used simultaneously: an inertial navigation system (INS) and an optical navigation system (ONS). Acetabular component anteversion and inclination were measured on a post-operative CT. *Results*: The average age of the patients was 64 years (range: 24–92) and the average BMI was 27 kg/m^2^ (range 19–38). 52% had hip surgery through an anterior approach. 98% of the INS measurements and 88% of the ONS measurements were within 10° of the CT measurements. The mean (and standard deviation) of the absolute difference between the postoperative CT and the intra-operative measurements for inclination and anteversion were 3.0° (2.8) and 4.5° (3.2) respectively for the ONS, along with 2.1° (2.3) and 2.4° (2.1) respectively for the INS. There was a significantly lower mean absolute difference to CT for the INS when compared to ONS in both anteversions (*p* < 0.001) and inclination (*p* = 0.02). *Conclusions*: We found that both inertial and optical navigation systems allowed for adequate acetabular positioning as measured on postoperative CT, and thus provide reliable intraoperative feedback for optimal acetabular component placement.

Level of Evidence: Therapeutic Level II.

## Introduction

Total hip arthroplasty (THA) is a highly successful procedure for end-stage hip osteoarthritis. Accurate component alignment is critical to ensure satisfactory postoperative outcomes and to minimise complications [1, 2]. Malalignment of the acetabular component in particular may increase the risk of dislocation, wear, loosening, impingement and overall patient dissatisfaction [1, 3]. Lewinnek et al. proposed a “safe-zone” of acetabular alignment in 1978 [4]. Despite this, several authors have reported that many dislocations still occur within that same safe zone [5, 6].

Currently, the vast majority of acetabular components are aligned manually [2]. The positioning of the acetabular component is estimated using anatomical landmarks such as the transverse acetabular ligament. However, patient characteristics such as obesity or muscularity can limit exposure and compromise cup positioning [7]. Positioning of the acetabular component is also greatly influenced by the patient’s pelvic position during surgery. During a THA, the pelvis can be tilted or shifted in any direction due to instability of the patient’s pelvis on the table or force placed on tissue retractors. This can happen even in the presumably more stable supine position for an anterior approach to the hip [7–9]. Furthermore, there are multiple studies demonstrating that manual acetabular component placement can have a high rate of malpositioning [10]. Callanan et al. found that only 50% of acetabular components were positioned within 10 degrees of the accepted range of abduction (30°–45°) and version (5°–25°) [10]. Thus to optimise acetabular alignment, surgical guidance technology has been increasingly used. It was found that the acetabular component anteversion and inclination were significantly more accurate in the computer-assisted group than in the manual-placed group [11]. Agarwal et al. also reported a reduced rate of revision for dislocation with the use of navigation-assisted THA.

Several types of navigation systems are commercially available and are proven to increase accuracy [11–15]. Optical navigation systems (ONS) involve the use of a mobile tower and navigation software to assist surgeons with the accuracy and consistency of prosthesis positioning. While its effectiveness in improving implant positioning has been widely demonstrated, there are several limitations to its use [15]. Traditionally, these computer navigation systems involve the use of bulky equipment, with variable reproducibility in registering anatomical landmarks, particularly if the patient is obese or if they are in the lateral position. The system needs a line of sight between the tracker in the operative field and the optical receptor, which is frequently blocked by the position of the operative assistant.

The use of an inertial navigation system (INS) for THA has been an area of increasing interest. There are multiple advantages to this type of navigation tool. Firstly, costs may be reduced compared to optical navigation systems as there are no substantial initial capital costs, and due to its compact sizing, there are fewer storage considerations. The system can also use the table as a reference, have a shorter set-up time and also not require an extensive patient registration process. As it does not rely heavily on landmark acquisitions for measurements, inaccuracies in certain patients as also reduced.

Therefore, this study aimed to compare the accuracy of two different types of navigation systems (ONS vs INS) for acetabular component alignment. This was done by comparing intra-operative measurements of inclination and anteversion with the gold standard post-operative CT measurements.

## Material and methods

### Patient selection

We prospectively included patients consecutively between June 1, 2020, and August 30, 2021, undergoing primary total hip arthroplasty or hip resurfacing arthroplasty at a single institution. Patients were eligible for inclusion if both navigation tools were simultaneously utilised during the operation and they received postoperative CT scans of the hip. Patients were excluded if one of the navigation systems was not used during the procedure, or they did not undergo a post-operative CT scan. An anterior approach to the hip was not used for obese patients or those undergoing hip resurfacing. The primary outcome was the position (anteversion and inclination) of the acetabular component on the hip being operated on. All relevant ethics approval and informed consent were obtained for patients included in this study.

### Procedure

Preoperatively a patient-specific optimal acetabular position was calculated from a preoperative CT scan and Electro-Optical System (EOS) lateral imaging of the lumbar spine, pelvis and hips in standing, relaxed seated and flexed seated position. Acetabular components were inserted in the precalculated position using both an INS and an ONS. Acetabular component implantation was performed by an experienced arthroplasty surgeon. At the time of acetabular component impaction, both navigation tools were separately mounted on the impactor handle and the final acetabular orientation was recorded. Demographic data collected included patient age, gender, BMI, surgical approach (direct anterior or posterolateral), type of implant and fixation (cemented or uncemented) used. The ultimate cup position was inserted using the INS for all patients.

### Inertial Navigation System

The INS (Navbit Sprint) is a single-use computer-assisted surgical navigation system containing rate gyroscopes and accelerometers to generate real-time information in regard to inclination and anteversion angles. To use the device, it is first registered in either of two positions – supine or lateral, with alignment guides provided for each position. For supine registration, the patient is placed in the supine position with the mount rigidly fixed to any position on the patient’s pelvis by means of two bone pins. A gravity vector is acquired which represents the functional anteroposterior axis. The surgical table is then tilted 10 degrees to the left and then 10 degrees to the right. The INS acquire two further gravity vectors at each tilted position: the perpendicular vector to these two vectors is the roll axis and this represents the functional longitudinal axis of the patient, thereby creating a functional coordinate system for calculation of the inclination and anteversion angles. For lateral registration, the initial gravity vector represents the functional transverse axis of the patient and the roll axis represents the longitudinal axis of the patient.

### Optical Navigation System

The ONS (the Stryker Navigation System mobile tower, in combination with the OrthoMap Versatile Hip Software) is a widely available system that is used for hip arthroplasty. This system requires tracker fixation onto the pelvic bone using the same surgical pins as the INS. For registration in the supine patient, the table is moved up and down. Ten points are collected representing the functional anteroposterior axis of the patient. The surgeon then digitises the left and right anterior superior iliac spines to create an anatomic transverse axis. Thereby creating a coordinate system that is partially functional and partially anatomic. For patients in the lateral position, the table is also moved up and down. Ten points are collected now representing the functional transverse axis of the patient. Two further points are then collected: a mid-axis lateral thoracic point and the greater trochanter. These two points define a functional longitudinal axis. The acetabular cup position is referenced from these coordinate systems.

### Measurement of navigation system

The ONS data were all anatomical measurements and recalculated to provide radiographic measurements according to the formulas postulated by Murray in 1993 [16]. The INS data collected are already radiographic measurements. A postoperative CT scan was performed to evaluate the acetabular component position. Early postoperative CT scans may be impacted by the pelvic roll or tilt caused by patient positioning, thus pelvic position was corrected using multiplanar reformation to ensure accurate measurement. The coronal CT images were used to measure the cup inclination. The inclination angle was measured from a line between the two teardrops and the peripheral margins of the cup. This provided radiographic data for cup inclination. The transverse images of the CT scan were used to measure the anteversion. This angle was formed from the line between the two infero-posterior borders of the ischium and the peripheral margins of the cup. This provided anatomical data for cup anteversion. The anteversion angles were then recalculated to provide radiographic data.

### Data analysis

The mean positions and the mean absolute difference of the INS and ONS data compared to CT measurements were calculated, together with their minimum, maximum and standard deviations. We calculated the percentage of cup positions within 10 degrees difference of the CT measurements. A Student’s t-test was used to compare the two systems with the level of significance set at 0.05. RedCap secure application was used for the registration of all patient- and operative data.

## Results

### Patient demographics

All consecutive patients recruited during the study period met the inclusion criteria. A total of 98 consecutive patients were included in this study, with 5 undergoing bilateral hip arthroplasty, resulting in 102 hips for analysis (52 anterior and 50 posteriors). The mean age of the patients at the time of surgery was 64 years (range: 24–92). For those undergoing an anterior approach, the mean age was 70 years (range: 45–92) compared to 59 years (range: 24–86) for those undergoing a posterior approach. Those undergoing an anterior approach all received THA, while those undergoing a posterior approach, received either hip resurfacing (94%) or THA (6%). The mean BMI of all patients was 27 kg/m^2^.

### Accuracy of inertial navigation

**Figure 1:**
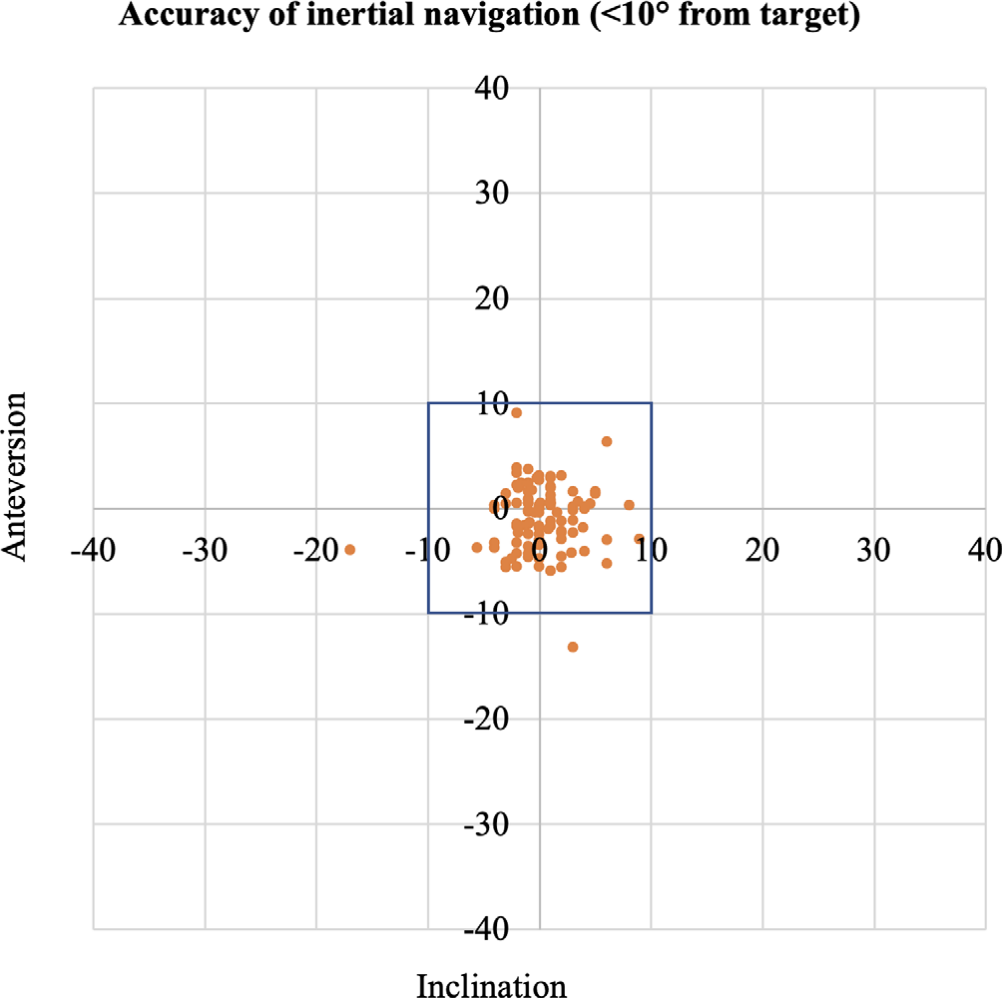
Accuracy of inertial navigation (<10° from target).

Two hips (2%) using INS had a mean absolute error greater than 10 degrees from the CT measurements. Patients with an anterior approach had 100% within 10 degrees of the target, which dropped in the posterior approach to 96% (Figure 1). The mean absolute error of the INS compared to the CT was 2.4° for anteversion and 2.1° for inclination (Table 1). For the patients undergoing an anterior approach, the absolute error of the INS compared to the CT was 2.0° for anteversion and 1.9° for inclination. For patients undergoing a posterior approach, the absolute error of the INS compared to the CT was 2.7° for anteversion and 2.3° for inclination.

**Table 1:** Mean absolute difference of CT measurements and each navigation system.

	CT measurements		Mean absolute difference vs INS		Mean absolute difference vs ONS	
	Inclination	Anteversion	Inclination	Anteversion	Inclination	Anteversion
Total	41.2 (3.5)	18 (3.6)	2.1 (2.3)	2.4 (2.1)	3.0 (2.8)	4.5 (3.2)
Anterior	41.7 (3.3)	19.5 (3.4)	1.9 (1.8)	2.0 (1.6)	3.0 (2.6)	3.6 (3.0)
Posterior	40.6 (3.6)	16.4 (3.1)	2.3 (2.8)	2.7 (2.5)	2.9 (3.1)	5.3 (3.3)

### Accuracy of optical navigation

**Figure 2:**
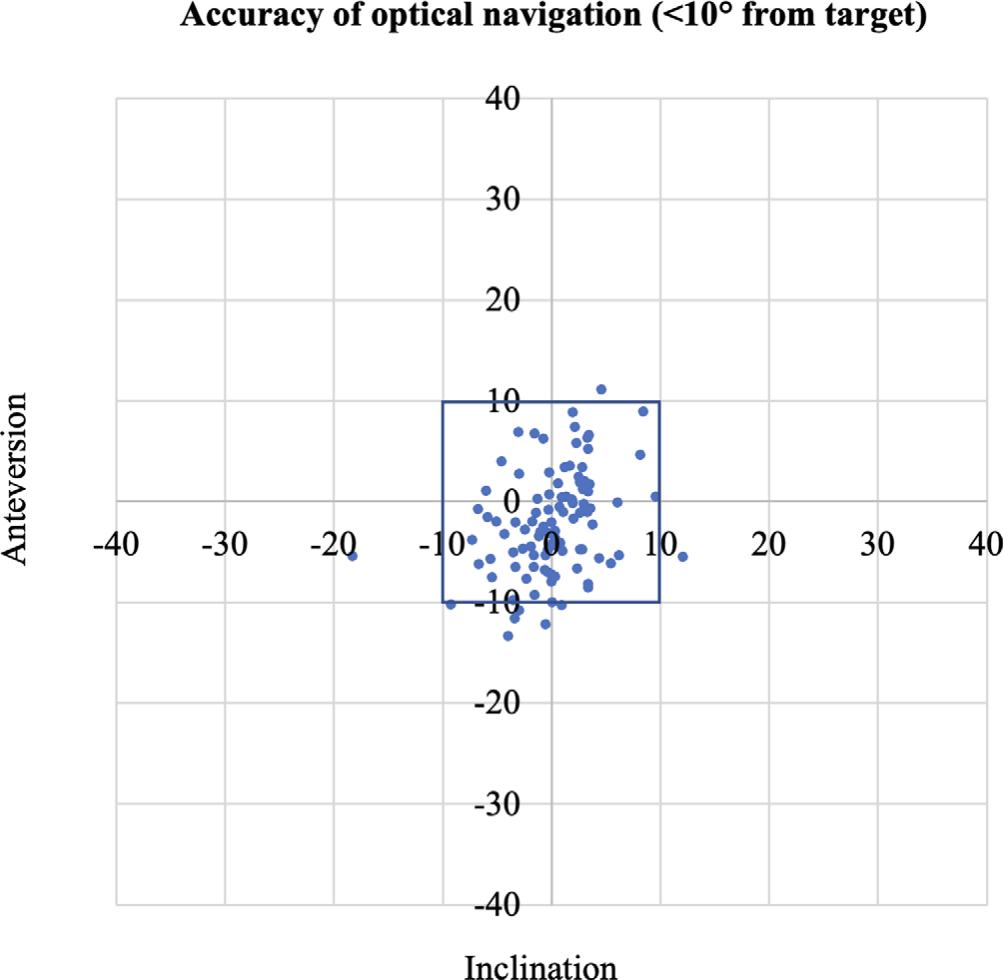
Accuracy of optical navigation (<10° from target).

Twelve hips (12%) using ONS had a mean absolute error greater than 10 degrees from the CT measurements. Patients with an anterior approach had 94% within 10 degrees of the target, which dropped in the posterior approach to 82% (Figure 2). The average absolute error of the ONS compared to the CT was 4.5° for anteversion and 3.0° for inclination (Table 1). For the patients undergoing an anterior approach, the absolute error of the INS compared to the CT was 3.6° for anteversion and 3.0° for inclination. For patients undergoing a posterior approach, the absolute error of the INS compared to the CT was 5.3° for anteversion and 2.9° for inclination.

### Accuracy of inertial vs optical navigation

There was a significantly lower mean absolute difference to CT for the INS when compared to ONS for both anteversion (*p* < 0.001) and inclination (*p* = 0.02). For patients undergoing an anterior approach, there was also a significantly lower mean absolute difference to CT for the INS when compared to ONS for both anteversion (*p* < 0.001) and inclination (*p* = 0.02). However, in the patients undergoing a posterior approach, the mean absolute difference to CT for the INS when compared to ONS was only significantly lower for anteversion (*p* < 0.001) but not inclination (*p* = 0.32).

## Discussion

This study aimed to assess the accuracy of optical and inertial navigation systems when compared to postoperative CT measurements of acetabular component positioning. The INS and ONS achieved 98% and 88% accuracy within 10 degrees of the CT measurement of anteversion and inclination. We demonstrated that both types of navigation produced reliable and reproducible acetabular cup positioning.

One of the major contributors to early revision following THA is the malposition of the acetabular cup [17]. A large cohort study found that only 50% of acetabular components were placed within 10 degrees of the optimal position even in the hands of an experienced surgeon [10]. The use of navigation during surgery can optimise cup positioning and reduce the number of early revisions and complications due to potentially avoidable reasons [10]. One of these complications is prosthetic hip dislocation, which is lower in navigation-assisted hip arthroplasties [2]. Despite this, only 4% of THA in Australia are currently implanted with navigation assistance [2].

Several studies have reported improved acetabular component positioning with the use of any type of navigation technique [11–15]. However many also noted that this optimised positioning was not associated with any significant effect on short-term outcomes such as early dislocation or pain [11–15]. The absence of significant clinical benefit associated with navigated THA may be explained by suboptimal planning of cup position. Navigation increases the accuracy of placement, which would result in the acetabular component being implanted in its intended position. For most studies, this optimal position is defined as the Lewinnek safe zone. However, as the safe zone is unsafe in other studies, this may suggest that we are aiming at the wrong targets [5, 6]. Studies combining patient-specific alignment and navigation could therefore provide further evidence of the benefit of this combination of factors.

The scatterplots assessing the accuracy of INS and ONS (Figures 1 and 2) highlight that these navigation systems still result in a few outliers. In particular, one hip was noted to incline both INS and ONS to be nearly 20 degrees different from the CT measurements. This patient underwent a hip resurfacing procedure through a posterior approach in the standard lateral decubitus position. Pre-operative assessment of this patient noted significant hip stiffness with limited abduction. We postulate that during the pre-operative disinfection routine, abduction of the leg may have tilted his pelvis due to his restricted abduction. Subsequently, both INS and ONS systems may have taken the tilted pelvis as the neutral plane during calibration, which would have resulted in a consistent inclination error across both systems but not affected the anteversion. This outlier emphasizes the importance of patient positioning on the operative table, and in particular, achieving a neutral pelvis position before any calibration of the navigation system.

It is important to acknowledge the limitations of this study. The number of patients is relatively small and there is no associated clinical follow-up data. While the “gold standard” of measuring anteversion and inclination is via a CT, there is still a risk of measurement error in calculating the final acetabular component position. We found comparable accuracy for both the anterior and posterior approach groups in our results. However, with the posterior approach group, there is potential for greater heterogeneity in the baseline patient characteristics as it consisted of two subgroups of patients. First, it included patients eligible for THA but with a relative contraindication for an anterior approach such as obesity. However, it also consisted of patients undergoing hip resurfacing arthroplasty, which tended to be patients who were younger, more likely to be male and had a lower BMI. This is in contrast to the anterior approach group, in which all patients received a THA. This study also did not compare the accuracy of cup placement using no navigation.

## Conclusions

This study is the first to report a prospective comparison between two different types of navigation tools for intraoperative cup positioning. Although we cannot comment on the long-term reduction of complications, we found that both navigation systems led to accurate cup positioning. This assistance with cup positioning can provide benefits over any free-hand technique, especially in patients with an altered acetabular structure (i.e. severe dysplasia) or extensive acetabular bone loss. Further large prospective randomised studies comparing manual and navigation-assisted THA according to a patient-specific alignment could further evaluate the value of navigation-assisted THA.
